# 

**Published:** 1914-12-15

**Authors:** 


					﻿the
DENTAL
REGISTER
Edited by DrNELVILLE S. HOFF
ANN ARBOR, MICH.
VOL. LXVIII. DECEMBER 1914 NO. 12
THE SAMUEL A. CROCKER CO., Publishers
OHIO DENTAL AND SURGICAL DEPOT
CONRAD BLDG., 18 & 20 W. Seventh St., CINCINNATI, O.
PRICE, ONE DOLLAR A YEAR
Entered at the Post-Office at Cincinnati, O , as second-class mall matter
				

